# Proteolytic processing of QSOX1A ensures efficient secretion of a potent disulfide catalyst

**DOI:** 10.1042/BJ20130360

**Published:** 2013-08-09

**Authors:** Jana Rudolf, Marie A. Pringle, Neil J. Bulleid

**Affiliations:** *Institute of Molecular, Cellular and Systems Biology, College of Medical Veterinary and Life Sciences, Davidson Building, University of Glasgow, Glasgow G12 8QQ, U.K.

**Keywords:** disulfide formation, proprotein convertase, proteolytic processing, quiescin sulfhydryl oxidase 1 (QSOX1), sulfhydryl oxidase, BMH, 1,6-bismaleimidohexane, CHO, Chinese-hamster ovary, DDM, dodecylmaltoside, DMEM, Dulbecco’s modified Eagle’s medium, EndoH, endoglycosidase H, ER, endoplasmic reticulum, Ero1, endoplasmic reticulum oxidase 1, GAPDH, glyceraldehyde-3-phosphate dehydrogenase, GM130, *cis*-Golgi matrix protein of 130 kDa, NEM, *N*-ethylmaleimide, PDI, protein disulfide-isomerase, PNGase, peptide N-glycosidase, PPC, proprotein convertase, QSOX, quiescin sulfhydryl oxidase, SEC, size-exclusion chromatography, TBST, TBS containing 0.1% Tween 20, TCA, trichloroacetic acid, TM, transmembrane

## Abstract

QSOX1 (quiescin sulfhydryl oxidase 1) efficiently catalyses the insertion of disulfide bonds into a wide range of proteins. The enzyme is mechanistically well characterized, but its subcellular location and the identity of its protein substrates remain ill-defined. The function of QSOX1 is likely to involve disulfide formation in proteins entering the secretory pathway or outside the cell. In the present study, we show that this enzyme is efficiently secreted from mammalian cells despite the presence of a transmembrane domain. We identify internal cleavage sites and demonstrate that the protein is processed within the Golgi apparatus to yield soluble enzyme. As a consequence of this efficient processing, QSOX1 is probably functional outside the cell. Also, QSOX1 forms a dimer upon cleavage of the C-terminal domain. The processing of QSOX1 suggests a novel level of regulation of secretion of this potent disulfide catalyst and producer of hydrogen peroxide.

## INTRODUCTION

QSOX1 (quiescin sulfhydryl oxidase 1) is an enzyme that can introduce disulfides into a wide range of proteins using molecular oxygen as an electron acceptor. It is a chimaera of two protein families as it contains both a disulfide-exchange (thioredoxin-like) and an oxidase (Erv-like) domain [1]. Its mechanism of action is well characterized *in vitro*, involving an initial disulfide exchange between the thioredoxin domain and substrate, and an internal electron transfer leading to the reduction of FAD to FADH_2_ within the Erv domain [2,3]. The FADH_2_ rapidly reduces molecular oxygen to liberate hydrogen peroxide. Hence, for every disulfide introduced into a protein substrate, one oxygen and hydrogen peroxide molecule is consumed and produced respectively. The reaction catalysed by QSOX1 is equivalent to that catalysed by a combination of Ero1 (endoplasmic reticulum oxidase 1) and PDI (protein disulfide-isomerase) in the ER (endoplasmic reticulum). However, the catalytic efficiency of QSOX1 is several orders of magnitude greater than Ero1, giving rise to the possibility that QSOX1 may have a role in catalysing disulfide formation in the ER [4]. Such a role has been given more credence by the observation that, when overexpressed, human QSOX1 can complement a yeast strain deficient in Ero1 activity [5]. However, although QSOX1 is synthesized in the ER, its subcellular location seems to be primarily in the Golgi apparatus [5], which may preclude any function in the ER.

**Figure 1:**
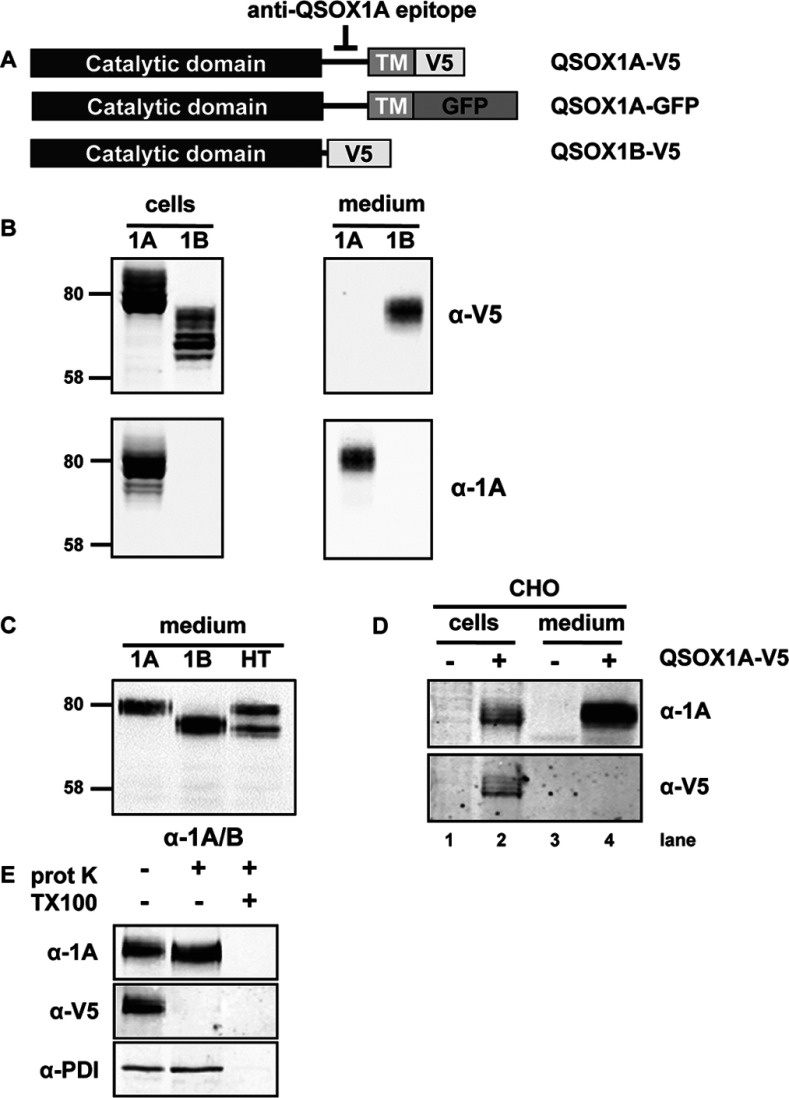
Expression and secretion of human QSOX1A (**A**) The QSOX1 constructs used in the present study are shown. Upper panel: the QSOX1A sequence including the C-terminal single-pass TM domain followed by a V5-tag (V5). The epitope detected by the anti-QSOX1A antibody is located on a linker that connects the Erv and TM domain. Middle panel: the same as above, only the C-terminal V5 tag is changed to a GFP tag. Lower panel: V5-tagged QSOX1B. (**B**) Western blots showing the expression and secretion of QSOX1A–V5 (1A) and QSOX1B–V5 (1B). Cell lysates were prepared from HT1080 cells stably overexpressing V5-tagged forms of either QSOX1A or QSOX1B. The secretion of proteins was allowed for 3 h into serum-free medium followed by TCA precipitation. Nitrocellulose membranes were co-probed for V5 (secondary DyLight680) and QSOX1A (secondary DyLight800). Molecular masses of marker proteins are indicated (in kDa). (**C**) Secretion of overexpressed forms of QSOX1A–V5 (1A), QSOX1B–V5 (1B) and endogenous QSOX1 forms (HT) is shown. Secretion of QSOX1 into serum-free medium was allowed for 3 h and proteins were affinity-purified using concanavalin A–Sepharose. Gel loading was adjusted according to the estimated secretion levels of QSOX1 from different cell lines. Nitrocellulose membranes were probed using an antibody detecting both forms of QSOX1 (α-1A/B). Molecular masses of marker proteins are indicated (in kDa). (**D**) A CHO cell line stably expressing human QSOX1A–V5 (+) was compared to untransfected CHO cells (−). Cell lysates and medium were prepared and analysed as described in (**B**). (**E**) Proteinase K protection assay. Semi-permeabilized QSOX1A–V5-expressing cells were incubated in the presence (+) or absence (−) of proteinase K (prot K) and/or Triton X-100 (TX100). Nitrocellulose membranes were co-probed for the V5 and QSOX1A epitopes (see **B**) and PDI (α-PDI), an ER marker indicating integrity of the inner membranes.

There are two isoforms of QSOX expressed in mammalian cells: QSOX1 and QSOX2 [6]. In addition, QSOX1 exists in two splice variants: QSOX1A and QSOX1B (Figure 1A). QSOX1A contains an additional 143 amino acids at the C-terminus, including a TM (transmembrane) domain. When newly synthesized, QSOX1A is localized to the membrane fraction, indicating that the TM domain is functional [5]. QSOX1B is not membrane-associated and can be secreted from a variety of tissues and is present in most bodily fluids [7,8]. The relative roles of QSOX1A and QSOX1B have been difficult to determine because of the identical protein sequence of QSOX1B and part of the ectodomain of QSOX1A, which compromises antibody localization studies. It is most likely that they have the same enzymatic function, but may fulfil their role in different cellular and extracellular environments. Both QSOX1 and QSOX2 are expressed in most tissues, but QSOX2 is much less abundant than QSOX1 [9]. QSOX2 shares 35% identity with QSOX1A having the same overall domain structure and containing a TM domain that confers membrane localization [10]. The significance of the presence of the different QSOX isoforms is unclear, as is the identity of any physiological substrates.

Although the precise function of QSOX1 remains elusive, what is known is that the expression of the enzyme is elevated in certain disease states. QSOX1 was originally identified as a protein that is up-regulated during the transition of cells into quiescence [11]. The protein has also been shown to be elevated in serum following heart failure and can therefore act as a prominent biomarker for cardiovascular disease [12]. In addition, QSOX1 expression is highly up-regulated during prostate tumorigenesis [13,14] and breast cancer [15], suggesting a role for this protein in cancer progression.

Even though QSOX1A contains a TM domain, plasma from patients with adenocarcinoma of the pancreas were found to contain a peptide that could only be derived from QSOX1A, suggesting that the protein is proteolytically cleaved during carcinogenesis [16]. To investigate the potential processing of QSOX1A, we studied its trafficking when expressed in mammalian cells. Our results demonstrate that the protein is initially membrane-associated, but is efficiently cleaved and secreted. In addition, the secreted processed protein exists as a dimer. QSOX1A and QSOX1B are capable of associating within cells, which could potentially prevent secretion of QSOX1B until QSOX1A is proteolytically cleaved. These observations provide compelling evidence that the enzyme is functional as a soluble protein outside the cell and provide an indication of how the trafficking and function of both splice variants may be regulated by intracellular proteolytic processing.

## EXPERIMENTAL

### Antibodies

The anti-QSOX1A antibody was raised in rabbit using the peptide EPPEHMAELQRNEQEQPL and affinity-purified. A rabbit anti-QSOX1 antibody that recognizes both QSOX1A and QSOX1B was a gift from Professor Debbie Fass (Weizmann Institute of Science, Rehovot, Israel). Rabbit anti-PDI antibody was as described previously [17] and the mouse anti-PDI antibody (1D3) was a gift from Professor David Vaux (University of Oxford, Oxford, U.K.). Mouse anti-MHC class I (HC10) [18] and rabbit anti-GM130 (*cis*-Golgi matrix protein of 130 kDa) antbodies were gifts from Dr Adam Benham (University of Durham, Durham, U.K.) and Professor Martin Lowe (University of Manchester, Manchester, U.K.) respectively. The remaining antibodies were purchased from the following companies: mouse anti-GAPDH (glyceraldehyde-3-phosphate dehydrogenase) (Ambion), rabbit anti-actin (Sigma), mouse anti-giantin (Abcam), mouse anti-V5 and anti-V5–agarose (Invitrogen), rabbit anti-V5 (Sigma) and rabbit anti-GFP (Thermo-Scientific). The following fluorescent-conjugated secondary antibodies were purchased: FITC-conjugated anti-rabbit and anti-mouse (Sigma), Texas Red-conjugated anti-rabbit and anti-mouse (Abcam), 680 and 800-conjugated anti-(mouse IgG) and anti-(rabbit IgG) (either Li-Cor IRDye or Thermo-Scientific DyeLight). Mouse anti-V5–agarose was purchased from Sigma and GFP-Trap®_A was from Chromotek.

### Cell lines and cell culturing

HT1080-based cell lines were cultured in DMEM (Dulbecco's modified Eagle's medium) (Gibco) and CHO (Chinese-hamster ovary) cells were grown in Ham's F12 medium (Sigma). Growth media were supplemented with 10% (v/v) FBS (Sigma) and penicillin/streptomycin (Gibco). The HT1080 and the CHO cell lines stably overexpressing QSOX1A-V5 were generated as described in [5]. The QSOX1A–GFP fusion was created by using the QSOX1A–V5/His construct as a template and cloning the entire sequence upstream of the V5-tag in the pEGFP vector (Clontech). QSOX1A–GFP was then subcloned into pcDNA3.1 Hygro (Invitrogen) for expression in mammalian cells. The same template was used to create the QSOX1B–V5 construct. Stable cell lines were created using polyethyleneimine-mediated transfection [19] (Polysciences).

### Deglycosylation

Cells were harvested, washed and lysed in lysis buffer [50 mM Tris/HCl, pH 8.0, containing 150 mM NaCl, 5 mM EDTA and 1% (w/v) Triton X-100] to give a concentration of 1.5×10^4^ cells/μl. Lysates were cleared by centrifugation at 20000 ***g*** for 10 min at 4°C, and reactions were set up following the manufacturer’s protocol (NEB). The samples were digested overnight at 37°C using 500 units of either EndoH (endoglycosidase H) or PNGase (peptide N-glycosidase) and separated by SDS/PAGE (7.5% gel).

### Membrane fractionation

For detection of soluble eGFP, HT1080 cells were transfected transiently with pCAsalEGFP [20] and cells were harvested after 18 h. HT1080 cells stably overexpressing QSOX1A–GFP were used for the detection of QSOX1A–GFP. Cells were washed with PBS and resuspended in 2 ml of homogenization buffer (50 mM Tris/HCl, pH 7.4, containing 250 mM sucrose, 50 mM KCl, 5 mM MgCl_2_, 1 mM EDTA, 0.5 mM PMSF and 1 mM DTT). Cells were homogenized by ten passes through a 12-μm clearance ball-bearing homogenizer (Isobiotec). Lysates were centrifuged at 1000 ***g*** for 2 min at 4°C, and the pellet, containing the nuclear fraction, was washed with 2 ml of homogenization buffer and stored on ice. The supernatant was centrifuged at 16000 ***g*** for 75 min at 4°C, and the pellet, containing the membrane fraction, was washed with 2 ml of homogenization buffer and stored on ice. The supernatant was precipitated with 10% (w/v) TCA (trichloroacetic acid) and 0.4 mg/ml deoxycholate, and the resulting pellet was washed with 80% (v/v) acetone. All pellets were resuspended in equal volumes of buffer A and analysed by SDS/PAGE (10% gel).

### Pulse–chase and immunoisolation of QSOX1A

Experiments were essentially carried out as described in [5]. In brief, cells were starved for 30 min in cysteine/methionine-free DMEM and then radiolabelled in the same medium containing EasyTag™ EXPRESS^35^S Protein Labeling Mix (Pierce) (0.4 MBq/ml). After 30 min of incubation at 37°C the radiolabel was removed, and cells were washed with PBS and incubated in complete DMEM (containing 0.5 mM cycloheximide) for various lengths of time. At specific time points, the medium was removed, centrifuged at 250 ***g*** for 5 min to remove contaminating cells and transferred to a fresh tube containing Protease Inhibitor Cocktail (Roche) and sodium azide to a final concentration of 0.02%. Cells were washed with PBS, before being lysed in RIPA buffer (50 mM Tris/HCl, pH 7.5, containing 150 mM NaCl, 1% Nonidet P40, 0.5% deoxycholate and Roche protease inhibitor cocktail). Cell debris was removed by centrifugation at 20000 ***g*** for 3 min at 4°C. The lysates and the medium were pre-cleared by adding Protein A–Sepharose (Generon) and incubated for 30 min at 4°C. Samples were subjected to immunoisolation by using anti-V5–agarose, GFP-Trap®_A or Protein A–Sepharose and anti-QSOX1A. Samples were incubated at 4°C either for 2 h (V5 and GFP) or overnight (QSOX1A) on a roller table. The Sepharose beads were pelleted by centrifugation at 800 ***g*** for 1 min and washed three times with 1 ml of RIPA buffer. An equal volume of SDS sample buffer (100 mM Tris/HCl, pH 6.8, containing 200 mM DTT, 4% SDS, 0.1% Bromophenol Blue and 20% glycerol) was added, and the samples were boiled for 10 min before separation by SDS/PAGE (8% gel for QSOX1A–V5 and 11% gel for QSOX1A–GFP). Gels were fixed, dried and exposed to phosphor plate or imaging film (Kodak BioMax MR film).

### Concanavalin A purification of secreted QSOX1

HT1080 cells stably overexpressing QSOX1A–V5 or QSOX1B–V5 and untransfected cells were incubated with serum-free medium for 3 h. The medium was harvested, contaminating cells removed by centrifugation at 250 ***g*** for 5 min, and protease inhibitor cocktail and sodium azide were added. The samples were pre-cleared with Protein A–Sepharose (30 min at 4°C) before being incubated in the presence of 20 μl of concanavalin A–Sepharose 4B (Sigma) and divalent metal ions (1 mM MgCl_2_, 1 mM MnCl_2_ and 1 mM CaCl_2_) for 16 h at 4°C on a roller table. Concanavalin A–Sepharose beads were isolated by centrifugation at 800 ***g*** for 1 min and washed three times with 1 ml of RIPA buffer. The volume of SDS sample buffer added was adjusted according to the estimated expression levels of the QSOX1 in these different cell lines. Finally, the samples were boiled and equal volumes were analysed by SDS/PAGE (11% gel).

### Immunoblotting

After separation by SDS/PAGE, proteins were transferred on to nitrocellulose membranes (Li-cor Biosciences). Membranes were blocked in 3% (w/v) non-fat dried skimmed milk powder in TBST (TBS containing 0.1% Tween 20) and incubated for 16 h at 4°C in the presence of primary antibodies. Membranes were incubated with the secondary fluorescent-conjugated antibodies for 45 min in TBST. Western blots were visualized on an Odyssey® SA IR scanner.

### Immunofluorescence and live-cell microscopy

Cells were grown on 13-mm-diameter coverslips (Thermo-Scientific) and immunostained as described previously [21]. After fixing with ice-cold methanol (5 min), cells were blocked in 1% (w/v) BSA in PBS for 45 min, followed by 30 min incubations each for the primary and then secondary antibody in 0.2% BSA in PBS at 20°C. Slides were mounted in Mowiol® (Calbiochem) containing 25 μg/ml DABCO (1,4-diazadicyclo[2.^2^.^2^]octane) (Sigma). Images were taken either with a LSM Pascal Exciter or a LSM 510 Meta confocal microscope (Zeiss). For live-cell microscopy, cells were grown in glass-bottomed culture dishes (35-mm-diameter Petri dish/10-mm-diameter microwell, MatTek Corp.), washed extensively with Hepes buffer (20 mM Hepes, pH 7.4, containing 130 mM NaCl, 5 mM KCl, 1 mM CaCl_2_, 1 mM MgCl_2_ and 10 mM d-glucose) and imaged on a LSM 510 Meta confocal microscope in Hepes buffer.

### Proteinase K protection assay

Semi-permeabilized cells were prepared as described in [22]. Proteinase K was added to a final concentration of 0.25 μg/ml to 1.5×10^5^ cells in KHM buffer (20 mM Hepes, pH 7.2, containing 110 mM potassium acetate and 2 mM MgCl_2_). Where appropriate, 1% (v/v) Triton X-100 was added. Reaction mixtures were incubated on ice for 30 min and stopped by the addition of 200 μl of PBS containing 1 mM PMSF and 2 mM EGTA. Samples were centrifuged at 250 ***g*** for 5 min, supernatant was removed, and the pellets were resuspended in lysis buffer. Reactions were cleared by centrifugation at 20000 ***g*** for 5 min at 4°C and analysed by SDS/PAGE (11% gel).

### BMH (1,6-bismaleimidohexane) cross-linking

BMH (Thermo-Scientific) was made up fresh in DMSO. Cells were detached with trypsin and washed with PBS, and cell pellets were resuspended in KHM buffer. BMH was added to the cell suspensions to a final concentration of 0.1 mM and samples were incubated on ice for 30 min. Reactions were stopped by the addition of 20 mM DTT for 5 min. Cells were pelleted at 250 ***g*** for 5 min at 4°C and lysed in lysis buffer and samples were cleared by centrifugation at 20000 ***g*** for 5 min at 4°C.

### SEC (size-exclusion chromatography)

Cell were grown in 225 cm^2^ flasks until confluent, detached with trypsin and washed with PBS. The cell pellets were lysed in gel-filtration buffer (50 mM Tris/HCl, pH 7.5, containing 150 mM NaCl and 1 mM EDTA) and 2% (w/v) DDM (dodecylmaltoside). Cell debris was removed by centrifugation at 20000 ***g*** for 10 min at 4°C, and a buffer exchange was carried out using Vivaspin centrifugal concentrators (molecular mass cut-off 50 000 Da) into gel-filtration buffer containing 0.02% DDM. For secreted protein, a 225 cm^2^ flask of confluent cells was incubated with 20 ml of serum-free DMEM for 3 h. The medium was then removed, centrifuged at 250 ***g*** for 5 min to remove contaminating cells and subjected to a buffer exchange in the same fashion as the cell lysates. For SEC, a Sephadex 200 PC3.2/30 (GE Healthcare) column was equilibrated in gel-filtration buffer containing 0.02% DDM. Then, 50 μl of each of the concentrated samples was loaded and 25 μl fractions were collected. Volumes of 18 μl of each fraction were mixed with SDS sample buffer and analysed by Western blotting. Western blots were quantified using ImageJ (NIH).

### QSOX1 co-assembly

HT1080 cells were transfected with equal amounts of QSOX1A–V5 and QSOX1A–GFP or QSOX1B–V5 and QSOX1A–GFP. After 24 h, the medium was removed, and free thiols were blocked by incubation with 20 mM NEM (*N*-ethylmaleimide) (Sigma) in PBS for 2 min on ice. After the cells were rinsed with PBS to remove residual NEM, they were lysed by addition of RIPA buffer. The cell lysates were processed as described above using GFP-Trap®_A. The samples were analysed by SDS/PAGE (8% gel) and immunoblotted using anti-GFP and anti-V5 antibodies.

## RESULTS

### QSOX1A is proteolytically processed and secreted from mammalian cells

QSOX1 has been shown previously to locate to the Golgi apparatus and to be secreted from mammalian cells [5,23]. As QSOX1A contains a TM domain, whereas QSOX1B does not, it was assumed that the localization pattern reflects that of membrane-associated compared with soluble protein. To assess the location of the two splice variants of QSOX1, we created stable cell lines expressing each protein tagged with a V5 epitope at the C-terminus (Figure 1A). Intracellular expression of the V5-tagged proteins was demonstrated for both QSOX1A and QSOX1B (Figure 1B, left-hand panel) with multiple products being present, probably reflecting different glycosylated forms. We also detected V5-tagged QSOX1B, but not V5-tagged QSOX1A, in the medium. An antibody raised against a peptide only present in QSOX1A (Figure 1A) was able to detect this protein not only in the cell lysate, but also surprisingly in the medium (Figure 1B, right-hand panel). The appearance of QSOX1A in the medium was not due to the overexpression of the protein, as we could isolate and detect both QSOX1A and QSOX1B from the medium of untransfected cells (Figure 1C, HT). We were able to detect both forms in similar amounts by first isolating glycoproteins from the medium of untransfected cells using concanavalin A, followed by immunoblotting using an antibody that recognizes both forms of the protein. Finally, we showed that the secretion of QSOX1A was not cell-line-specific, as QSOX1A was also processed and secreted following expression in CHO cells (Figure 1D). Hence it would appear that both splice variants are secreted from mammalian cells. The absence of the V5 epitope from secreted QSOX1A suggests that the protein becomes proteolytically processed within the cell, although the different mobility of the secreted QSOX1A and QSOX1B forms shows that the processing does not generate QSOX1B from QSOX1A. Our results also demonstrate that similar amounts of both isoforms are expressed and secreted at least by HT1080 cells.

We have shown previously that QSOX1A does become integrated into the ER membrane when expressed in an *in vitro* translation system in the presence of a source of ER membranes [5]. To determine whether the protein also becomes integrated into the membrane in intact cells, we treated semi-permeabilized QSOX1A–V5-expressing cells with proteinase K. In the absence of protease, QSOX1A–V5 was detected by both the anti-QSOX1A and the anti-V5 antibodies (Figure 1E). In the presence of protease, the V5 epitope was removed, whereas the QSOX1A epitope remained, demonstrating that the protein was integrated into the membrane with the V5 epitope exposed to the protease on the cytosolic side of the membrane. PDI was used as a control ER-localized protein that, like QSOX1A, was only digested after proteinase K digestion in the presence of detergent.

**Figure 2:**
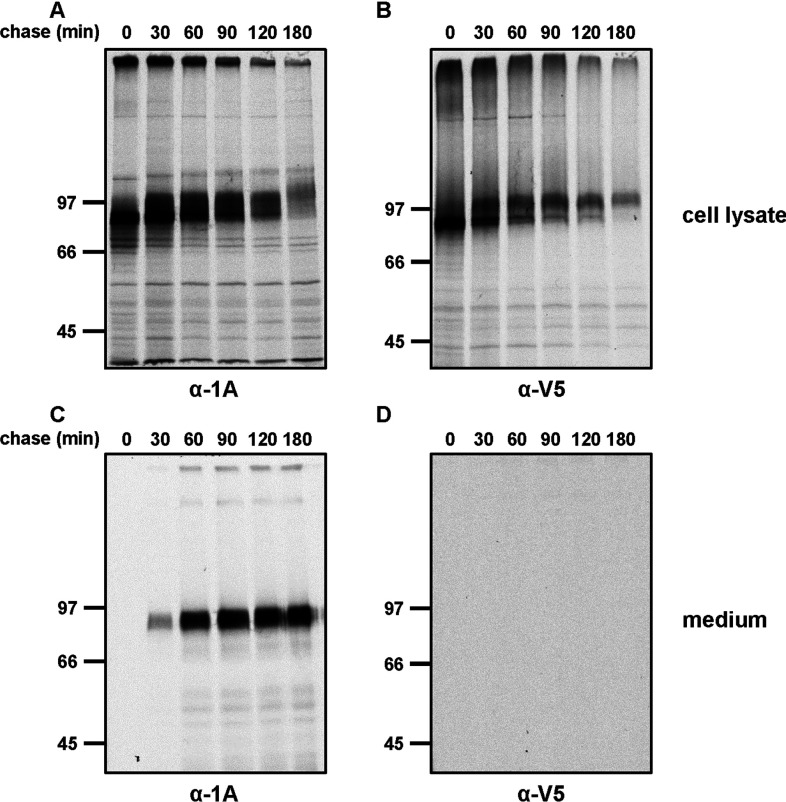
Pulse–chase experiments illustrating the secretion of QSOX1A The cells were radiolabelled for 30 min and secretion was followed by harvesting cells (**A** and **B**) and medium (**C** and **D**) at the indicated time points. Samples were immunoisolated by using either the anti-QSOX1A antibody bound to Protein A–Sepharose (α-1A) (**A** and **C**) or anti-V5-coupled agarose (α-V5) (**B** and **D**). The samples were separated by SDS/PAGE and gels were exposed to Kodak imaging film. Molecular masses of marker proteins are indicated (in kDa).

The accumulation of QSOX1A in the medium was clearly observed; however, these experiments do not provide any indication of the kinetics of secretion. To address this point, we carried out a pulse–chase experiment (Figure 2). Newly synthesized protein was radiolabelled for 30 min and then chased for various times before being immunoisolated with either the anti-QSOX1A or anti-V5 antibody. Intracellular QSOX1A migrates as a distinct band immediately after the pulse, which becomes progressively shifted to a slower migrating more diffuse band during the chase (Figures 2A and 2B). This shift is most likely to be due to processing of the oligosaccharide chains in the Golgi apparatus. The slower migrating QSOX1A is also detected by the anti-V5 antibody, suggesting that proteolytic processing does not occur until after the protein has reached the Golgi apparatus. Within 30 min of the chase, QSOX1A is detected in the medium by the anti-QSOX1A antibody, but not by the anti-V5 antibody, and is depleted from cells after 180 min, demonstrating the rapid processing and secretion of this protein (Figures 2C and 2D).

### Subcellular location of QSOX1A processing

**Figure 3:**
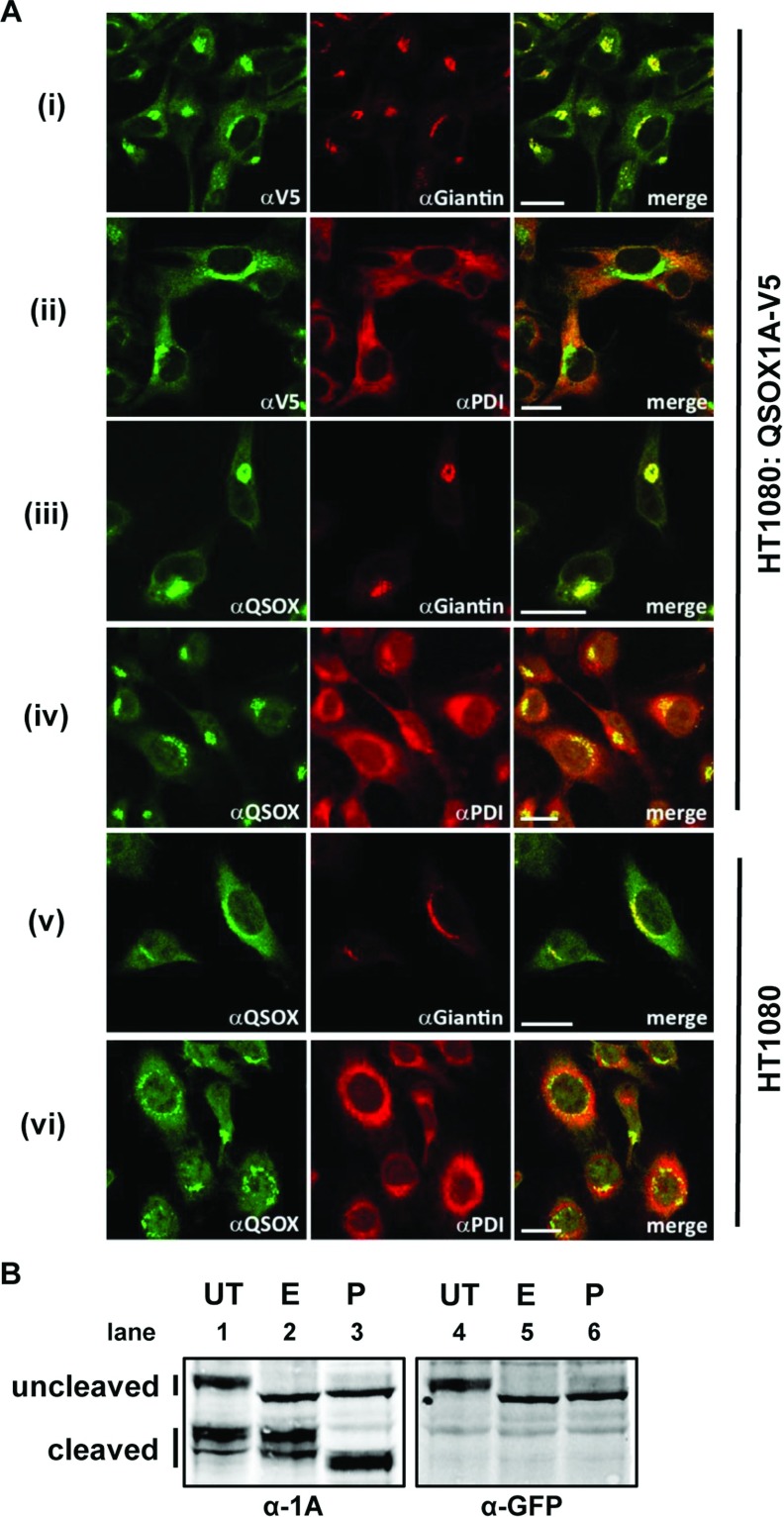
Intracellular localization of QSOX1A (**A**) Immunofluorescent microscopy images of fixed cells are shown. Panels (i)–(iv): localization of the QSOX1A–V5 form stably overexpressed in HT1080 cells. Panels (v) and (vi): localization of endogenous QSOX1A in untransfected HT1080 cells. Cells were co-stained for (i) V5 (green)/giantin (red), (ii) V5 (green)/PDI (red), (iii) QSOX1A (green)/giantin (red), (iv) QSOX1A (green)/PDI (red), (v) QSOX1A (green)/giantin (red), (vi) QSOX1A (green)/PDI (red). Giantin and PDI are Golgi and ER markers respectively. Merged images are shown on the right and yellow colour indicates co-localization. Scale bar, 20 μm. (**B**) Deglycosylation experiments using the QSOX1A–GFP-expressing HT1080 cell line. Cell lysates (UT) were treated with either EndoH (E) or PNGase (P) overnight at 37°C. Samples were separated by SDS/PAGE, transferred on to a nitrocellulose membrane and probed for either QSOX1A (α-1A) or the GFP tag (α-GFP). Full-length (uncleaved) and cleaved products of QSOX1A are indicated.

To identify the location of the QSOX1A processing event, we first determined the subcellular location of the V5-tagged protein by immunofluorescence (Figure 3A). QSOX1A–V5 co-localized primarily with the Golgi protein giantin with some co-localization with the ER protein PDI (Figure 3A, panels i and ii). Very similar staining was seen with the anti-QSOX1A antibody (Figure 3A, panel iii and iv). In addition, we carried out immunofluorescent microscopy of the endogenous QSOX1A revealing a similar Golgi localization (Figure 3A, panels v and vi), suggesting that the accumulation of QSOX1A–V5 is not a consequence of overexpression. These results, along with the pulse–chase data, suggest that the V5-tagged protein is present in the Golgi apparatus and that proteolytic processing occurs after transport from the ER.

To investigate further the location of proteolytic processing, we created a stable cell line expressing QSOX1A containing a GFP tag at the C-terminus. Removal of the GFP tag caused a clear shift in mobility of the protein when separated by SDS/PAGE (Figure 3B, lane 1), allowing us to differentiate between the cleaved and uncleaved forms. Only the uncleaved form is recognized by an anti-GFP antibody (Figure 3B, lane 4) and is sensitive to digestion with both EndoH and PNGase (Figure 3B, lanes 5 and 6). However, the cleaved form shows resistance to digestion with EndoH, but not PNGase (Figure 3B, lanes 2 and 3). Modification of the oligosaccharide side chain that results in EndoH resistance occurs in the medial Golgi [24]. As our pulse–chase experiment demonstrated that cleavage does not occur until after some modification of the oligosaccharide side chain in the Golgi (Figure 2B), then these results allow us to conclude that cleavage is most likely to occur following transport from the ER but before trafficking to the medial Golgi.

### Proteolytic processing of QSOX1A occurs at multiple sites within the ectodomain

**Figure 4:**
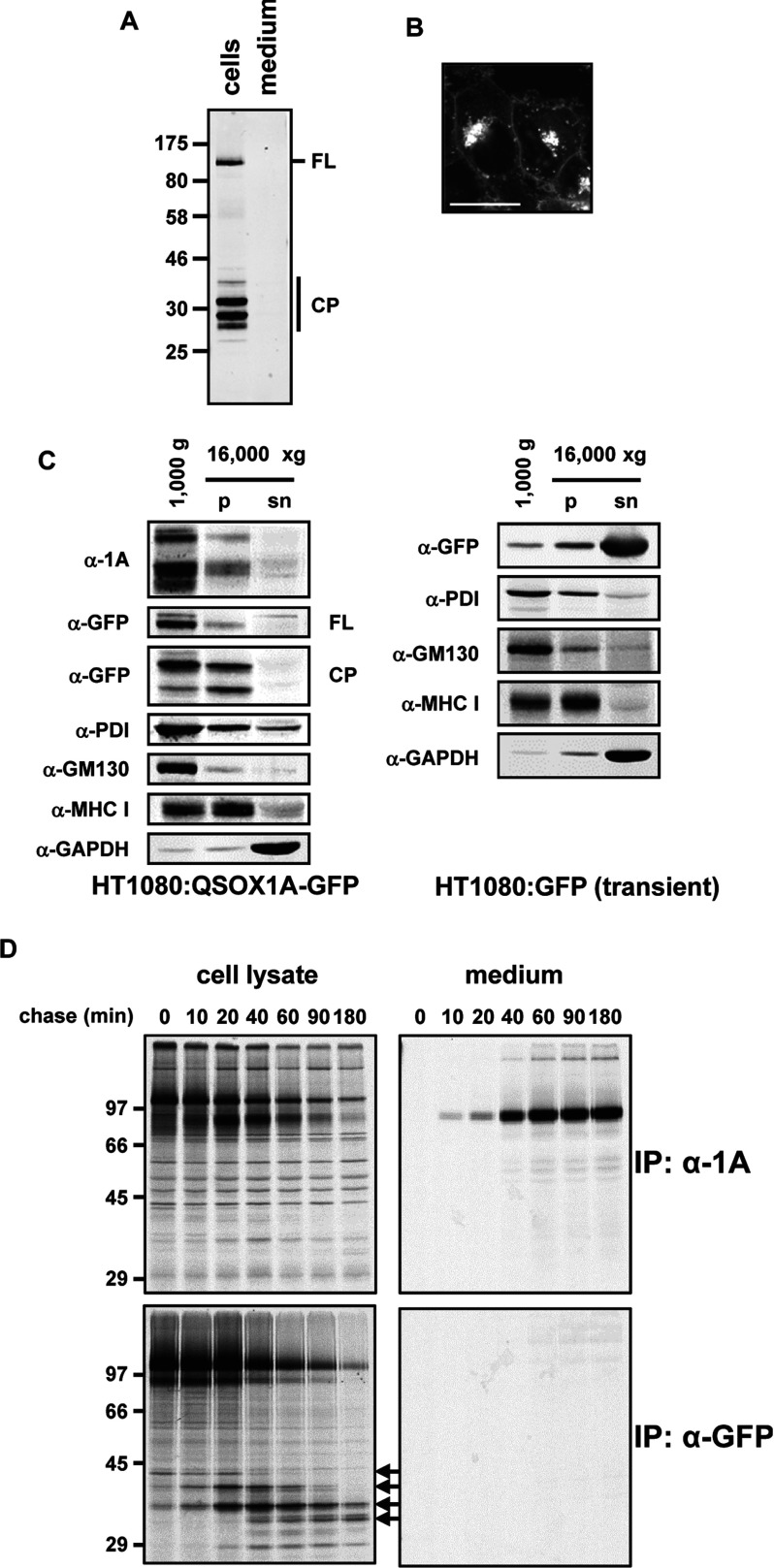
Cleavage products of QSOX1A–GFP (**A**) Western blot showing the full-length GFP-tagged form (FL) of QSOX1A and cleavage products (CP). The cell lysate and serum-free culture medium were harvested following 3 h of incubation in serum-free medium. The GFP-containing fragments were isolated using GFP-Trap®_A and identified by an anti-GFP Western blot. Molecular masses of marker proteins are indicated (in kDa). (**B**) Live-cell image of QSOX1A–GFP-expressing HT1080 cells by confocal microscopy. The GFP fluorescence is detected in the Golgi, the cell membranes and small vesicles. Scale bar, 20 μm. (**C**) Western blot analysis of the fractionation of cellular components by differential centrifugation. Cell lysates were homogenized and followed by two consecutive centrifugation steps at 1000 ***g*** and 16000 ***g***. The pellet of the first step contains nuclear membranes, the ER and the Golgi. The pellet (p) of the 16000 ***g*** centrifugation step enriches the cell membranes and the supernatant (sn) represents the cytoplasm. Nitrocellulose membranes were probed for cleaved and full-length forms of QSOX1A–GFP (α-1A), the GFP-tagged full-length form (α-GFP, FL) and cleavage products (α-GFP, CP), the ER marker PDI (α-PDI), the Golgi marker GM130 (α-GM130), a marker for the cell membrane (α-MHC I) and a cytoplasmic marker (α-GAPDH). Cell lysates were prepared from HT1080 cells stably expressing QSOX1A–GFP (HT1080:QSOX1A-GFP) and from HT1080 cells transiently transfected with soluble GFP (HT1080:GFP) respectively. (**D**) Pulse–chase experiments illustrating the cleavage of QSOX1A–GFP. The samples were radiolabelled for 30 min and secretion followed by harvesting cells and medium at the indicated time points. Samples were immunoisolated by using either the anti-QSOX1A antibody bound to Protein A–Sepharose or GFP-Trap®_A. The samples were separated by SDS/PAGE and gels were exposed to Kodak imaging film. Cleavage products are marked by arrows. Molecular masses of marker proteins are indicated (in kDa). IP, immunoprecipitation.

We used the cell line expressing GFP-tagged QSOX1A to characterize further the cleavage products. GFP-containing peptides were affinity-isolated from the cell lysate or medium (Figure 4A). Similarly to what we found with the V5-tagged protein, GFP-containing polypeptides were only isolated from the cell lysate and not from the medium. Several cleavage products could be isolated which migrated with apparent molecular masses greater than that of GFP alone which has a molecular mass of approximately 27 kDa. This result indicates that the cleavage site is not within GFP itself. The removal of the TM domain of QSOX1A could occur in two possible ways: either by regulated intra-membrane proteolysis or by cleavage in the ectodomain of the protein [25]. To distinguish between these possibilities, we carried out live-cell imaging of the QSOX1A–GFP cell line to determine where GFP fluorescence is localized in these cells. Bright intracellular punctate staining is seen indicative of a Golgi localization. In addition, the plasma membrane is clearly labelled (Figure 4B). As cleavage of QSOX1A occurs in the Golgi, this staining pattern would suggest that the GFP-containing cleavage products still contain the TM domain, are membrane-localized and have been transported to the cell surface.

To test this hypothesis, we carried out subcellular fractionation by differential centrifugation (Figure 4C). If QSOX1A had been cleaved at the cytosolic side of the membrane or within the TM domain, then the cleavage product should be fractionated with the cytosol. However, both the cleaved QSOX1A and the cleavage products which contain the GFP tag are present in the membrane fractions and co-fractionate with ER and Golgi proteins (Figure 4C, left-hand panel). As a control, we demonstrated that, when GFP alone is expressed in HT1080 cells, it co-fractionates with the cytosolic marker protein GAPDH (Figure 4C, right-hand panel). Finally, when the two major affinity-purified GFP-containing cleavage products were excised from the gel and digested with trypsin, peptides corresponding to the TM domain and the C-terminal region of the ectodomain were identified by MS (results not shown). Taken together, these results strongly suggest that QSOX1A cleavage occurs within the ectodomain of the protein while the TM domain remains intact.

The large shift in mobility between QSOX1A–GFP and cleaved QSOX1A allowed us to monitor the kinetics of cleavage and secretion in more detail. A pulse–chase analysis revealed processing of QSOX1A–GFP within the first 20 min of the chase, presumably following its transport from the ER to the Golgi (Figure 4D, upper panel). Cleaved QSOX1A appears in the medium shortly after intracellular processing, demonstrating rapid secretion from the Golgi. The GFP-containing products also appear at the same time, with the larger product seen first, followed by the small products at later time points, consistent with sequential cleavage events (Figure 4D, lower left-hand panel). No radiolabelled products were immunoisolated with the GFP-Trap®_A from the medium, confirming the absence of secretion of the uncleaved protein or the cleavage products.

### Identification of the cleavage site

**Figure 5:**
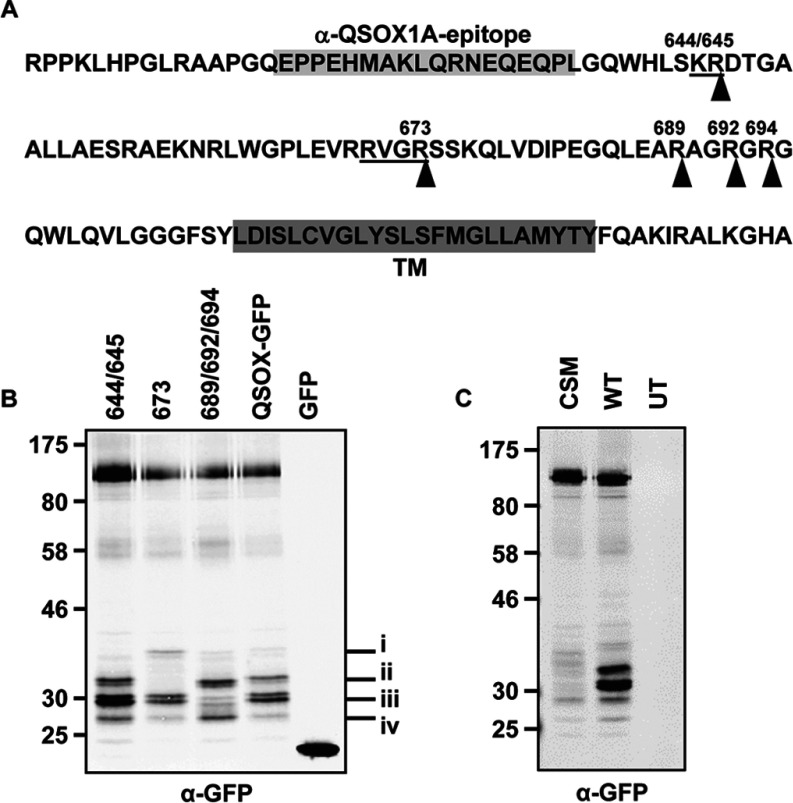
Identification of the cleavage sites in QSOX1A–GFP (**A**) Residues Arg^605^–Ala^742^ of QSOX1A are shown. The peptide sequence used to raise the anti-QSOX1A antibody is highlighted in light grey and the TM domain is highlighted in dark grey. Predicted PPC motifs are underlined; residues where cleavage occurs are numbered and the position of the incision is indicated with a triangle. (**B**) Western blot of QSOX1A–GFP full-length and cleavage products (i–iv) of different alanine mutants are shown. The GFP-containing peptides were isolated using GFP-Trap®_A and identified by an anti-GFP (α-GFP) Western blot. The positions of the respective alanine mutations are shown above the gel. QSOX-GFP, not mutated; GFP, soluble GFP. (**C**) Samples were processed as in (**B**) from HT1080 cells that were either untransfected (UT) or transfected with QSOX1A–GFP or a cleavage site mutant (CSM) where Arg^644^, Arg^645^, Arg^673^, Arg^689^ and Arg^694^ were mutated to alanine. Molecular masses of marker proteins are indicated (in kDa).

The results above define the location of the cleavage site in QSOX1A as occurring between the peptide sequence used to raise the anti-QSOX1A antibody and the TM domain (Figure 5A). To identify potential proteases responsible for the cleavage of QSOX1A, we first searched the UniProt database [26] for all proteases that are known to be present within the human ER and Golgi apparatus (Supplementary Table S1 at http://www.biochemj.org/bj/454/bj4540181add.htm). The resulting list of proteases was analysed manually with regard to their substrate specificities and consensus cleavage patterns. Three of the PPCs (proprotein convertases), PCSK3, PCSK6 and PCSK7, particularly stood out as they cleave at dibasic motifs, two of which are present in the QSOX1A amino acid sequence. The QSOX1A amino acid sequence also was analysed for potential cleavage sites using the ProP 1.0 Server [27]. Two of the predicted PPC cleavage sites are present in the region of interest with cleavage occurring C-terminally of residues Arg^645^ and Arg^673^ (Figure 5A). In addition, a third potential site that contains a consensus site for cleavage by the PPC family (i.e. K/R-X*_n_*-K/R where *n*=0, 2, 4 or 6 and X is any amino acid) [28], is present (Arg^689^–Arg^692^). To determine whether these sites are indeed used to process QSOX1A, we carried out mutagenesis using the QSOX1A–GFP construct as a template. Following transfection into HT1080 cells, we monitored the appearance of the cleavage products. In agreement with earlier observations (Figure 4D), cleavage does not occur at a single site. The K644A/R645A mutant led to the disappearance of a minor product (Figure 5B, i). The cleavage product (Figure 5B, ii) is a result of incision after Arg^673^. Furthermore, a triple mutation of Arg^689^, Arg^692^ and Arg^694^ led to the reduction of additional cleavage products (Figure 5B, iii). Preventing the cleavage at Arg^689^/Arg^692^/Arg^694^ gave rise to a faster migrating cleavage product barely seen with the wild-type protein (Figure 5B, iv). When multiple PPC motifs were mutated (Arg^644^, Arg^673^, Arg^689^, Arg^692^ and Arg^694^), none of the major cleavage products were seen (Figure 5C), although a small amount of cleaved material was secreted (results not shown). Hence no single mutation prevented cleavage, indicating that multiple sites may be recognized by the protease(s) and that each site is recognized independently. Taken together, these results suggest multiple, but specific, PPC cleavage sites within QSOX1A which result in the release of the ectodomain from its membrane anchor.

### QSOX1A forms a complex before proteolytic processing

**Figure 6:**
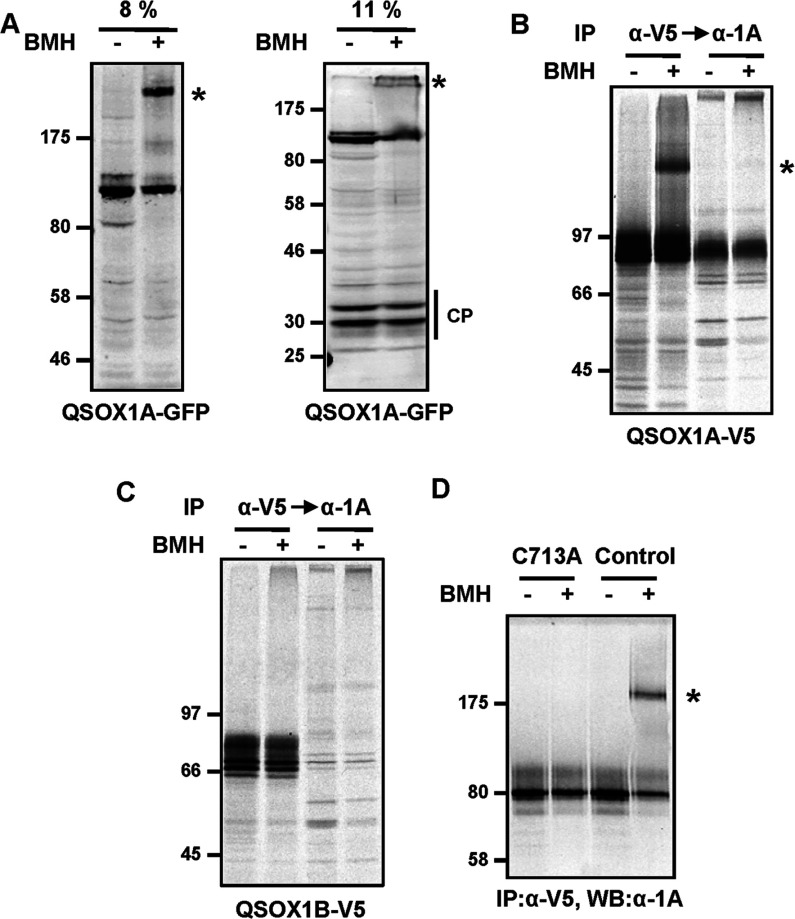
Dimerization of full-length QSOX1A Thiol-cross-linking experiments are shown in the presence (+) or absence (−) of 0.1 mM BMH. (**A**) Cell lysates were prepared from the QSOX1A–GFP-overexpressing cell line and analysed by Western blotting following separation by SDS/PAGE (8% and 11% gels respectively). Cleavage products (CP) are as indicated. (**B** and **C**) Proteins were labelled for 30 min in ^35^S-containing medium, and cells were harvested after 30 min of chase and subjected to BMH treatment as indicated. Cell lysates from a QSOX1A–V5- (**B**) and a QSOX1B–V5- (**C**) overexpressing cell line were first depleted of V5-containing polypeptides using anti-V5–agarose (α-V5). V5-immunodepleted lysates were then subjected to immunoisolation with anti-QSOX1A (α-1A). (**D**) Thiol-cross-linking was carried out as described in (**A**) using HT1080 cell lines expressing the QSOX1A–V5 C713A mutant form (transient) or the QSOX1A–V5 control (stable). Following immunoisolation with anti-V5–agarose (α-V5), proteins were detected in a Western blot using the anti-1A (α-1A) antibody. In (**A**), (**B**) and (**D**), the cross-linked product is indicated by an asterisk. IP, immunoprecipitation; WB, Western blot. Molecular masses are indicated (in kDa).

The synthesis of QSOX1A as a precursor protein that becomes cleaved may provide a mechanism for the regulation of protein function. To determine whether there was any effect of proteolytic processing on the interaction of QSOX1A with itself or with other proteins in cells, we stabilized interactions using a thiol-specific cross-linking agent (Figure 6). Using the QSOX1A–GFP-expressing cell line, a higher-molecular-mass product was detected using the anti-GFP antibody that was apparent only following cross-linking (Figure 6A). This result indicates the presence of uncleaved QSOX1A–GFP in complex either with itself or with other protein(s). When the cell lysates were separated on a higher percentage gel, no cross-linking of the GFP cleavage products was observed. This suggests that the ectodomain is required for stabilizing the cross-link or that the cysteine residues involved in the cross-linking are not present in the GFP cleavage products.

We also carried out cross-linking experiments using the QSOX1A–V5 cell line. For this, cellular proteins were radiolabelled and cross-linked, and cell lysates were subjected to immunoisolation, first with the anti-V5 antibody, then the V5-immunodepleted lysates were subjected to immunoisolation with the anti-QSOX1A antibody (Figure 6B). The anti-V5 antibody only detects the uncleaved QSOX1A–V5 form. In accordance with results described above, a product was isolated that was only present following cross-linking. In addition, the lysate depleted of V5-reactive proteins does not contain the cross-linked product, demonstrating further that only the uncleaved QSOX1A–V5 forms a complex. Finally, when the QSOX1B–V5 cell line was analysed in a similar experiment, no cross-linked products were seen following immunoisolation with the anti-V5 antibody (Figure 6C). This result implies that the appearance of a distinct cross-linked product is a specific property of uncleaved QSOX1A. Taken together, these results indicate that QSOX1A forms a complex with itself or with another protein before cleavage.

To identify the specific residues involved in the thiol-specific cross-linking of QSOX1A, we constructed a series of cysteine mutants. The only mutation that affected the cross-linking was C713A which is present within the TM domain of QSOX1A (Figure 6D). Hence the TM domain must be in close proximity to another QSOX1A molecule or other membrane proteins before cleavage. The absence of cross-linking of the cleavage products that contain Cys^713^ underlines the fact that the ectodomain is required for a stable association of the TM domain. Furthermore, analysis of the cross-linked product by MS revealed only the presence of peptides present in QSOX1A (results not shown). These results suggest that the cross-linked product is a dimer of QSOX1A.

**Figure 7:**
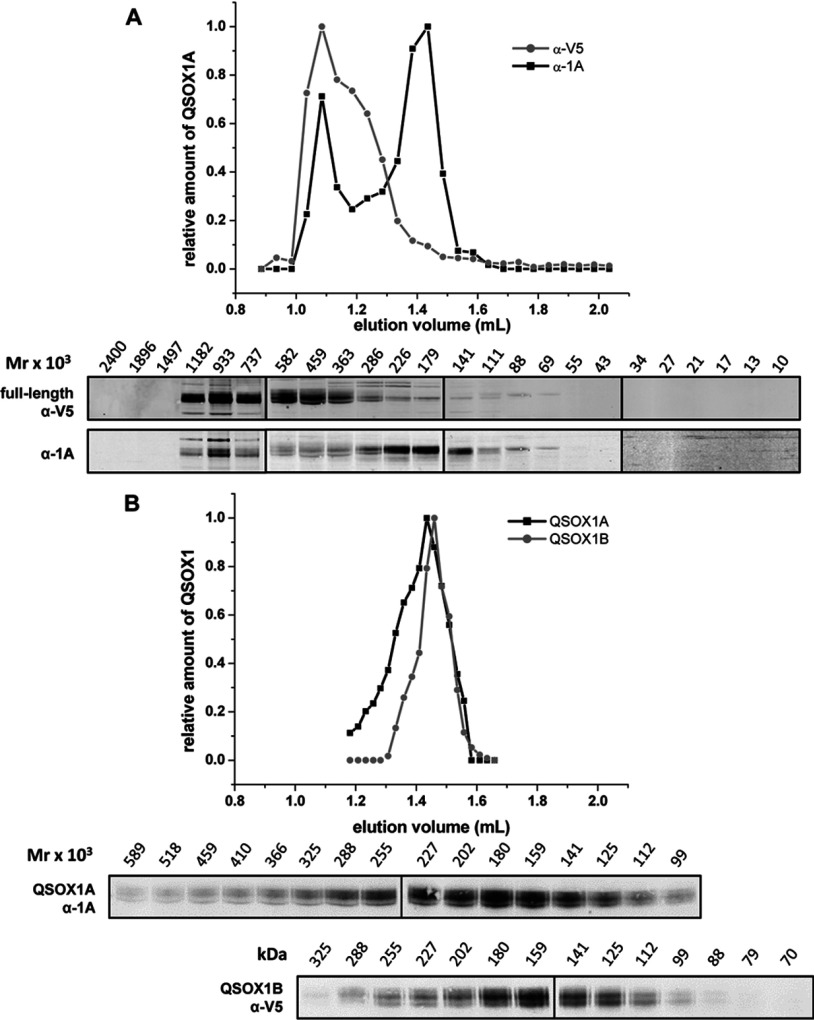
Analytical SEC of QSOX1 Samples were separated on a S200 PC3.2/30 SEC column (GE Healthcare) and fractions were analysed by Western blotting. (**A**) Cell lysate from a QSOX1A–V5-overexpressing cell line was prepared in gel-filtration buffer containing 0.02% DDM. Upper panel: normalized Western blot quantification results (relative amount of QSOX1A) are plotted against the SEC elution volume (ml). Each data point represents a fraction. Lower panel: Western blots of SEC fractions were co-probed with anti-V5 (α-V5) and anti-QSOX1A (α-1A) antibodies. Average molecular mass (Mr × 10^3^) for each fraction is indicated above the gels. (**B**) QSOX1 secreted into serum-free medium from cell lines stably expressing QSOX1A–V5 and QSOX1B–V5 respectively. The medium was removed from cells after 3 h and a buffer exchange was carried out into gel-filtration buffer containing 0.03% Triton X-100 using Vivaspin 50 000 Da molecular-mass cut-off concentrators. The samples were concentrated and analysed as described above.

The cross-linking approach allowed us to evaluate the association of QSOX1A in intact cells, but tells us little about the size of any complexes formed. To provide an alternative approach to determine complex formation, we separated cell lysates from the QSOX1A–V5-expressing cell line by SEC (Figure 7A). There was a clear separation of the uncleaved V5-tagged protein and the cleaved QSOX1A. Both were eluted as relatively broad peaks with the cleaved material eluting as a dimer (~180 kDa), whereas the uncleaved protein eluted as higher-molecular-mass material (~600–1200 kDa). SEC of the secreted QSOX1A or QSOX1B revealed the presence of a peak equivalent to a dimer and with no higher-molecular-mass material (Figure 7B). From these results and the cross-linking results, we can conclude that uncleaved QSOX1A forms a complex within cells before the formation of a dimeric species upon cleavage.

**Figure 8:**
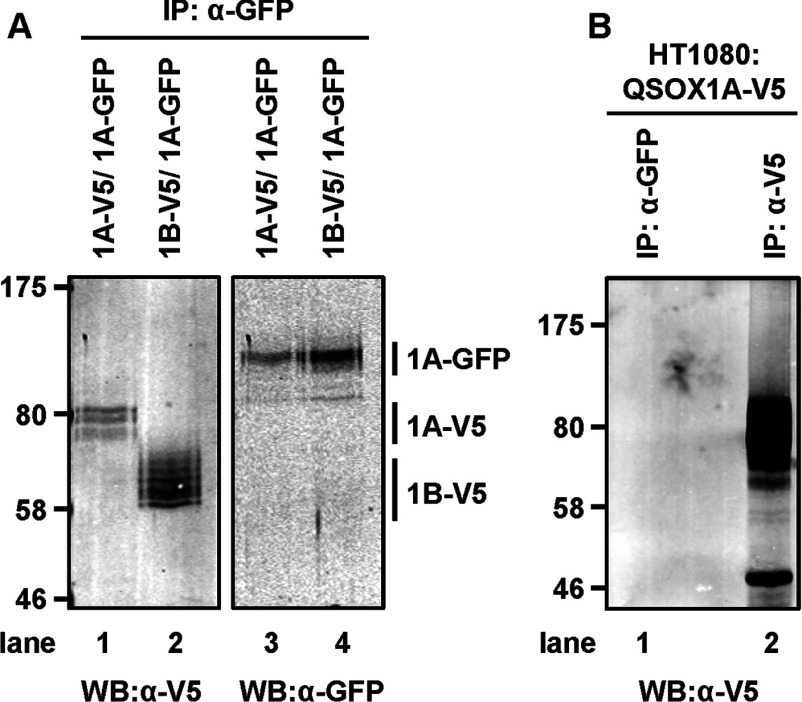
Interactions between QSOX1A and QSOX1B forms (**A**) HT1080 cells were co-transfected either with QSOX1A–V5/QSOX1A–GFP or QSOX1B–V5/QSOX1A–GFP as indicated above the blots. Cell lysates were subjected to anti-GFP (α-GFP) immunoisolation followed by Western blot analysis identifying either V5- or GFP-tagged QSOX1 forms as indicated on the right-hand side. (**B**) The control experiment shows that QSOX1A–V5 does not interact non-specifically with the GFP-Trap®_A resin. The lysates from a QSOX1A–V5-overexpressing cell line was incubated first with GFP-Trap®_A and then the immunodepleted lysate was subjected to immunoisolation with anti-V5–agarose (α-V5). Molecular masses of marker proteins are indicated (in kDa). IP, immunoprecipitation; WB, Western blot.

The ability of QSOX1A to form a dimer led us to consider the possibility that QSOX1B may be able to co-assemble with QSOX1A. To test this possibility, we carried out co-transfection of HT1080 cells with V5-tagged QSOX1B or V5-tagged QSOX1A and GFP-tagged QSOX1A. Following immunoisolation with the GFP-Trap®_A, we carried out immunoblotting to determine whether either V5-tagged QSOX1 isoform was co-isolated with QSOX1A–GFP (Figure 8). QSOX1A–V5 and QSOX1B–V5 were co-isolated, as judged by their presence in the immunoisolate (Figure 8A, lanes 1 and 2). As a control, we showed that QSOX1A–V5 could not be detected by immunoblotting following GFP-Trap®_A immunoisolation in the absence of QSOX1A–GFP (Figure 8B). These results indicate that QSOX1A can assemble not only with itself, but also with QSOX1B before cleavage of the TM domain, with the consequence that QSOX1B secretion could be dependent on the proteolytic cleavage of QSOX1A.

## DISCUSSION

Previous research has provided us with a detailed understanding of the enzymatic function and structure of the QSOX1 family of sulfhydryl oxidases [3,6], yet our knowledge of their function *in vivo* is limited. In the present study, we provide compelling evidence that both the short and long forms of QSOX1 are efficiently secreted from cells and that QSOX1A undergoes intracellular proteolytic processing. The efficiency of secretion would argue for an extracellular role for this enzyme as the presence of QSOX1 within the secretory pathway will be transitory. However, significant accumulation of the enzyme does occur in the Golgi apparatus and little, if any, QSOX1A staining is found at the cell surface. The accumulation of the enzyme in the Golgi would suggest that trafficking of QSOX1A to the cell surface requires the cleavage of the TM domain. Such regulated trafficking would provide a mechanism to control the level of enzyme released from cells.

Although the proteolytic cleavage of QSOX1A provides a way to regulate its secretion, no such restriction would be placed upon the secretion of QSOX1B. However, the ability of QSOX1A before its cleavage to interact with QSOX1B suggests a mechanism for controlling the secretion of QSOX1B. The interaction of QSOX1B with membrane-integrated QSOX1A would cause the secretion of both proteins to be dependent on the cleavage event. The ectodomain of QSOX1A is identical with that of QSOX1B apart from a C-terminal extension of approximately 104 amino acids in QSOX1A, so it is not too surprising that they can interact within the secretory pathway. Our analysis of the relative levels of secretion of these two forms of QSOX would suggest that they are secreted in approximately stoichiometric amounts at least from HT1080 cells. Hence the intracellular proteolytic processing of QSOX1A may well influence the concentration of both forms of this enzyme in the extracellular space.

Although it is apparent from our results that QSOX1A is cleaved within the Golgi, the exact identity of the protease(s) catalysing this event remains to be determined. Our mutagenesis studies have identified a number of sites for the incision that fit the consensus cleavage site for the PPC family [28]. This family of serine proteases cleaves proteins at basic sites during their passage through the secretory pathway. Some of the family members are resident in the Golgi and would be potential candidates for the QSOX1A protease. Previously, one member of the PPC family (PCSK5) was found to be co-immunoisolated from rat brain with a QSOX antiserum [29], although this enzyme seems to be poorly expressed in HT1080 cells [28]. We have carried out several experiments to try to inhibit cleavage with protease inhibitors, but none were able to completely prevent cleavage (results not shown). It may well be that several enzymes are capable of catalysing this cleavage event, precluding the use of inhibitors to identify the specific protease(s) involved.

The functional significance of the intracellular complex formation and membrane cleavage of QSOX1 is unclear, but it is tempting to speculate that enzymatic function could be regulated by subtle conformational changes occurring following proteolytic processing. QSOX1 is an efficient catalyst of disulfide formation with broad substrate specificity [30]. The fact that a product of the reaction of QSOX1 with protein thiols is hydrogen peroxide would suggest that the enzyme needs to be regulated to prevent excessive build-up of this reactive oxygen species [31]. Ero1 is tightly regulated by the formation of non-catalytic disulfides to prevent excessive hydrogen peroxide production. The non-catalytic disulfides prevent disulfide transfer to the substrate [32–34]. QSOX1 would appear to have no such regulatory mechanism and therefore, if unregulated, should be active both within and outside the ER during transit through the secretory pathway. It is known that soluble QSOX1 undergoes a dramatic conformational change during its enzymatic cycle [3]; tethering the enzyme to the membrane via a TM domain may well prevent or restrict such conformational changes leading to an inhibition of activity.

QSOX1 has been shown to introduce disulfides into a wide range of protein substrates; the only common characteristic is that the proteins need to be in a partially unfolded state [30]. Unlike the disulfide-exchange protein PDI, the thioredoxin domain of QSOX1 does not possess isomerase activity, indicating that the enzyme is equally proficient at introducing non-native as well as native disulfides [35]. Such an activity would restrict the yield of correctly folded protein as any non-native disulfide would not be resolved [31]. Within the ER, PDI could remove any non-native disulfides formed by QSOX1, providing an efficient mechanism for disulfide formation in newly synthesized proteins. Outside the ER, most proteins are likely to be folded correctly as they will have escaped the ER quality control [36]. Our identification of QSOX1 as an efficiently secreted catalyst of disulfide formation would suggest that its substrates are likely to be essentially folded, but require a catalyst to introduce regulatory disulfides to activate or inhibit protein function. Alternatively, QSOX1 may be required to stabilize multisubunit complexes by the formation of interchain disulfides.

## Online data

Supplementary data
