# Supplementary material for: Proteolytic processing of QSOX1A ensures efficient secretion of a potent disulfide catalyst
Source: Biochem J. 2013 Aug 9;454(Pt 2):181–90. doi: 10.1042/BJ20130360 (PMC3749868; doi:10.1042/BJ20130360)
Supplement: Supplementary data [file bj4540181add.pdf]

BJ Biomolecules

Biochem. J. (2013) **454**, 181–190 (Printed in Great Britain) doi:10.1042/BJ20130360

online data

SUPPLEMENTARY ONLINE DATA

Proteolytic processing of QSOX1A ensures efficient secretion of a potent disulfide catalyst

Jana RUDOLF*, Marie A. PRINGLE* and Neil J. BULLEID*[1]

*Institute of Molecular, Cellular and Systems Biology, College of Medical Veterinary and Life Sciences, Davidson Building, University of Glasgow, Glasgow G12 8QQ, U.K.

www.biochemj.org Biochemical Journal

Table S1 Human proteases present in the ER and Golgi

Results were returned upon a Uniprot database (http://www.uniprot.org) enquiry using the following search terms: Human/Protease/Golgi Stack [OR] Human/Protease/Endoplasmic Reticulum. Proprotein convertases are highlighted in bold.

Entry	Gene name	Protein names
P56817	*BACE1*	β-Secretase 1 (membrane-associated aspartic protease 2)
Q9Y5Z0	*BACE2*	β-Secretase 2 (membrane-associated aspartic protease 1)
A6NHC0	*CAN8*	Calpain-8
Q9Y646	*CBPQ*	Carboxypeptidase Q (plasma glutamate carboxypeptidase)
O60344	*ECE2*	Endothelin-converting enzyme 2
Q9NZ08	*ERAP1*	Endoplasmic reticulum aminopeptidase 1 (type 1 tumour necrosis factor receptor shedding aminopeptidase regulator)
Q6P179	*ERAP2*	Endoplasmic reticulum aminopeptidase 2
Q7Z2K6	*ERMP1*	Endoplasmic reticulum metallopeptidase 1
O75844	*FACE1*	CAAX prenyl protease 1 homologue (farnesylated protein-converting enzyme 1, zinc metalloproteinase Ste24 homologue)
**P09958**	***FURIN***	**Furin (paired basic amino acid residue-cleaving enzyme, PACE)**
Q92643	*GPI8*	GPI (glycosylphosphatidylinositol)-anchor transamidase
Q8TCT9	*HM13*	Minor histocompatibility antigen H13 (intramembrane protease 1, signal peptide peptidase)
Q92876	*KLK6*	Kallikrein-6
Q14703	*MBTP1*	Membrane-bound transcription factor site-1 protease (subtilisin/kexin isoenzyme 1, SKI-1)
O75900	*MMP23*	Matrix metalloproteinase-23
**P29122**	***PCSK6***	**Proprotein convertase subtilisin/kexin type 6 (paired basic amino acid cleaving enzyme 4, PACE4)**
**Q16549**	***PCSK7***	**Proprotein convertase subtilisin/kexin type 7 (proprotein convertase 7, PC7; proprotein convertase 8, PC8)**
P49768	*PSN1*	Presenilin-1 (PS-1)
P49810	*PSN2*	Presenilin-2 (PS-2)
P67812	*SC11A*	Signal peptidase complex catalytic subunit SEC11A
Q9BY50	*SC11C*	Signal peptidase complex catalytic subunit SEC11C
Q9Y6A9	*SPCS1*	Signal peptidase complex subunit 1 (12 kDa subunit)
Q15005	*SPCS2*	Signal peptidase complex subunit 2 (25 kDa subunit)
P61009	*SPCS3*	Signal peptidase complex subunit 3 (22/23 kDa subunit)
Q8TCT7	*SPP2B*	Signal peptide peptidase-like 2B (intramembrane protease 4)
Q8IUH8	*SPP2C*	Signal peptide peptidase-like 2C intramembrane protease 5)
Q8TCT6	*SPPL3*	Signal peptide peptidase-like 3 (intramembrane protease 2)
P57727	*TMPS3*	Transmembrane protease serine 3
Q9UI38	*TSP50*	Probable threonine protease PRSS50 (testis-specific)
O94966	*UBP19*	Ubiquitin C-terminal hydrolase 19
Q8NFA0	*UBP32*	Ubiquitin C-terminal hydrolase 32
P09936	*UCHL1*	Ubiquitin C-terminal hydrolase isoenzyme L1
Q9NUQ7	*UFSP2*	Ufm1-specific protease 2
Q96JH7	*VCIP1*	Deubiquitinating protein VCIP135

Received 13 March 2013/23 May 2013; accepted 28 May 2013
Published as BJ Immediate Publication 28 May 2013, doi:10.1042/BJ20130360

[1] To whom correspondence should be addressed (email neil.bulleid@glasgow.ac.uk).

© 2013 The Author(s)
The author(s) has paid for this article to be freely available under the terms of the Creative Commons Attribution Licence (CC-BY) (http://creativecommons.org/licenses/by/3.0/) which permits unrestricted use, distribution and reproduction in any medium, provided the original work is properly cited.
